# Reduced Skeletal Muscle Protein Turnover and Thyroid Hormone Metabolism in Adaptive Thermogenesis That Facilitates Body Fat Recovery During Weight Regain

**DOI:** 10.3389/fendo.2019.00119

**Published:** 2019-02-28

**Authors:** Julie Calonne, Laurie Isacco, Jennifer Miles-Chan, Denis Arsenijevic, Jean-Pierre Montani, Christelle Guillet, Yves Boirie, Abdul G. Dulloo

**Affiliations:** ^1^Department of Endocrinology, Metabolism and Cardiovascular System, Faculty of Sciences and Medicine, University of Fribourg Fribourg, Switzerland; ^2^Université Clermont Auvergne, INRA, UNH, Unité de Nutrition Humaine, CHU Clermont-Ferrand, Service de Nutrition Clinique, CRNH Auvergne Clermont-Ferrand, France; ^3^EA3920 and EPSI Platform, Bourgogne Franche-Comté Université Besançon, France

**Keywords:** thermogenesis, obesity, catch-up growth, thrifty metabolism, caloric restriction, deiodinase

## Abstract

**Objective:** The recovery of body composition after weight loss is characterized by an accelerated rate of fat recovery (preferential catch-up fat) resulting partly from an adaptive suppression of thermogenesis. Although the skeletal muscle has been implicated as an effector site for such thrifty (energy conservation) metabolism driving catch-up fat, the underlying mechanisms remain to be elucidated. We test here the hypothesis that this thrifty metabolism driving catch-up fat could reside in a reduced rate of protein turnover (an energetically costly “futile” cycle) and in altered local thyroid hormone metabolism in skeletal muscle.

**Methods:** Using a validated rat model of semistarvation-refeeding in which catch-up fat is driven solely by suppressed thermogenesis, we measured after 1 week of refeeding in refed and control animals the following: (i) *in-vivo* rates of protein synthesis in hindlimb skeletal muscles using the flooding dose technique of ^13^C-labeled valine incorporation in muscle protein, (ii) *ex-vivo* muscle assay of net formation of thyroid hormone tri-iodothyronine (T3) from precursor hormone thyroxine (T4), and (iii) protein expression of skeletal muscle deiodinases (type 1, 2, and 3).

**Results:** We show that after 1 week of calorie-controlled refeeding, the fractional protein synthesis rate was lower in skeletal muscles of refed animals than in controls (by 30–35%, *p* < 0.01) despite no between-group differences in the rate of skeletal muscle growth or whole-body protein deposition—thereby underscoring concomitant reductions in both protein synthesis and protein degradation rates in skeletal muscles of refed animals compared to controls. These differences in skeletal muscle protein turnover during catch-up fat were found to be independent of muscle type and fiber composition, and were associated with a slower net formation of muscle T3 from precursor hormone T4, together with increases in muscle protein expression of deiodinases which convert T4 and T3 to inactive forms.

**Conclusions:** These results suggest that diminished skeletal muscle protein turnover, together with altered local muscle metabolism of thyroid hormones leading to diminished intracellular T3 availability, are features of the thrifty metabolism that drives the rapid restoration of the fat reserves during weight regain after caloric restriction.

## Introduction

The recovery of body weight after substantial weight loss or diminished growth rate is accompanied by a high efficiency of fat deposition (1–7). This in part results from an adaptive suppression of thermogenesis which contributes to the preferential catch-up fat phenomenon (8) whereby body fat is recovered at a disproportionately faster rate than that of lean body mass. Such thrifty (energy conservation) metabolism for preferential catch-up fat probably had evolutionary survival value as it contributes to the rapid restoration of survival capacity conferred by the rapid recovery of the fat reserves in preparation for the next period of food scarcity. Nowadays, however, it is contributing to the “metabolic adaptation” that facilitates obesity recidivism after therapeutic slimming (9), and has also been implicated as a component of the “imprinted” thrifty phenotype in the link between early growth perturbations, excessive fat deposition during subsequent catch-up growth and later development of obesity and cardiometabolic diseases (10).

The effector mechanisms underlying this thrifty metabolism driving catch-up fat remain elusive. However, as the skeletal muscle is a major site for thermogenesis, glucose utilization and lipid oxidation, the possibility arises that a reduction in thermogenesis in this tissue could result in the redirection of spared fuel toward fat storage in adipose tissue (8). Which thermogenic effectors in skeletal muscle are suppressed to spare energy for catch-up fat are, however, far from being understood. A role for the uncoupling protein homologs UCP2 and UCP3—which have been proposed as potential uncouplers of oxidative phosphorylation (11)—is unlikely on the basis that their patterns of expressions in skeletal muscle do not fit with diminished thermogenesis in response to starvation and refeeding (12). By contrast, a number of findings suggest a role for altered peripheral action of the main active thyroid hormone, tri-iodothyronine (T3) for which skeletal muscle is a major target (13–15). Indeed, the circulating levels of T3, which is well-known to be diminished during caloric restriction, tend to remain lower (albeit marginally) in refed animals than in controls during the phase of catch-up fat (16, 17). More recently, it was shown that the net local synthesis of T3 in the gastrocnemius muscle, which is diminished during semistarvation, persists during the dynamic phase of catch-up fat, and is associated with several features of diminished intracellular availability of T3, in particular delayed contraction-relaxation kinetics and increased proportion of slow at the expense of fast muscle fibers (18). Taken together, these alterations in thyroid hormone-dependent properties of skeletal muscle constitute mechanisms that could underlie diminished skeletal muscle thermogenesis during weight loss and which persist during weight regain for the purpose of accelerating fat recovery.

As protein synthesis and protein turnover (an energetically costly substrate cycling) is also under the control of thyroid hormones, with protein turnover in skeletal muscle estimated to contribute to as much as 20% of whole body protein turnover (19–23), we investigated here (i) the extent to which the processes of protein synthesis and protein turnover may be diminished during the dynamic phase of catch-up fat in various muscle types varying widely in fiber composition, and (ii) their associations with altered skeletal muscle thyroid hormone metabolism and changes in the levels of the deiodinases (DIO1, DIO2, and DIO3) that modulate the local metabolism and intracellular availability of T3.

## Materials and Methods

### Animals

Sprague-Dawley male rats (Elevage Janvier, Le Genest-Saint-Isle, France), 6 weeks-old, were adapted to room and cage environments for 5–7 days prior to the start of each experiment. They were caged singly in a controlled room (22 ± 1°C) with a 12-h light-dark cycle, and maintained on a commercial pelleted chow diet (Provimi-Kliba SA, Kaiseraugst, Switzerland) consisting, by energy, of 24% protein, 66% carbohydrate, and 10% fat, and had free access to tap water. Animals were maintained in accordance with the regulations and guidelines of the Department of Medicine, University of Fribourg, for the care and use of laboratory animals; all experimental procedures were performed under conditions approved by the Ethical Committee of the State of Fribourg Veterinary Office.

### Experimental Design

Experiments were performed according to our previously reported design of semistarvation-refeeding that established a rat model for studying adjustments in energy expenditure specific for accelerating fat deposition during refeeding (3, 4, 16, 17). In all experiments, the semistarved rats were caloric restricted at 50% of *ad libitum* energy intake for 2 weeks, after which they were refed for periods of either 1 or 2 weeks, and comparisons made with control rats having similar body weight at the onset of refeeding. Both refed and control groups were provided with (and consumed) the same amount of a semisynthetic diet, which corresponded to that consumed during spontaneous food intake on pelleted chow; the details of composition of this semisynthetic (low-fat) diet and assessments of metabolizable energy (ME) intake have been reported previously (4).

### Body Composition Analysis

After the animals were killed by decapitation, the whole carcasses were dried to a constant weight in an oven maintained at 70°C and were subsequently homogenized for analysis of total fat content by the Soxhlet extraction method (24). The dry fat-free mass (dry FFM) was determined by subtracting total body fat and body water content from body weight, and the protein mass was calculated as follow: Protein mass (g) = dry FFM (g)^*^0.8; as detailed previously (4).

### Energy Balance and Energetic Efficiency Calculations

Energy balance measurements were conducted during refeeding by the comparative carcass technique over periods during which ME intake was monitored continuously, and energy expenditure over 2 weeks was determined as the difference between energy gain and ME intake. Body energy gain, fat gain, and protein gain during the 2 weeks of refeeding were obtained as the difference between the final and initial values (with the latter values estimated from values obtained from the group killed at the onset of refeeding). Total body energy content, and ΔBody energy can be calculated from a general formula relating the total energy value of the carcass, energy derived from fat, and energy derived from protein (4).

### Determination of Protein Turnover *in-vivo*

#### Tracer Administration

Protein synthesis rate was measured by incorporation of a stable isotope in the form of labeled amino acid (^13^C-valine) into the protein pool using the flooding dose method (25–27). Reagents were obtained from Sigma Chemical (St Louis, MO, USA) and L-[^13^C]-valine (99 atom percent excess) was obtained from Eurisotop France (Saint-Aubin, France). Muscle protein synthesis rates were assessed in hindlimb skeletal muscles by using the flooding-dose method. Food was removed early in the morning (07:00 h). At 6–7 h later, i.e., in the postabsorptive phase, rats were injected subcutaneously with L-[^13^C]-valine [300 μmol (100 g body)^−1^]. Fifty minutes after the injection of L-[^13^C]-valine (incorporation time), the animals were sacrificed, and skeletal muscles (gastrocnemius, soleus, tibialis anterior) were quickly excised, weighed, frozen in liquid nitrogen and stored at −80°C until further analyses. The contralateral muscles were also dissected intact, blotted and weighed, and frozen in liquid nitrogen for later total protein determination by the method of Lowry (28).

#### Analytical Method

Muscle samples (50 mg) were homogenized in an ice-cold buffer using Polytron homogenizer (PT1200C, Kinematica, Switzerland). After precipitation of the homogenate, centrifugations, and protein hydrolysis, amino acids were derivatized, and measurements of L-[^13^C]-valine enrichment in hydrolyzed proteins were performed by gas chromatography-combustion-isotope ratio mass spectrometry (Gas system, Fisons Instruments, VG isotech, Middlewich, UK). L-[^13^C]-valine enrichments in tissue fluid were assessed using gas chromatography-mass spectrometer (GC-MS) (HP5890, Hewlett-Packard, Paris, France) and used as precursor pool enrichment for the calculations of the fractional synthesis rates.

#### Calculations of Fractional Synthesis Rate (K_syn_)

This is calculated as previously described (25, 26). Basal subgroups (*n* = 4) are used for the determination of natural isotopic abundance in proteins in the muscles, as follows: Fractional synthetic rate (K_syn_) = (Ei × 100)/(Ep × t), where Ei represents the enrichment as atom percentage excess of [^13^C] derived from valine in muscle proteins at time t (minus basal enrichment); Ep is the mean enrichment in the precursor pool (tissue fluid L-[^13^C]-valine); t is the incorporation time (from time of tracer injection to sacrifice) expressed per day; data on K_syn_ are expressed as percentage per day (%/d).

#### Calculations of Fractional Growth Rate (K_growth_)

For each tissue, K_growth_ (expressed as %/d) is determined as the average K_growth_ over 48 h immediately before the measurement of protein synthesis as described by Samuels et al. (29), and is calculated as follows:

K_growth_ = (Δbody mass/Δt) × (Δtissue protein mass/Δbody mass) × (100/tissue protein mass), where
(i) (Δbody mass/Δt) is the body growth rate of individual animals during the 48 h before measurement of protein synthesis,
(ii) (Δtissue protein mass/Δbody mass) is the x-coefficient of a linear regression of tissue protein mass against body weight of all animals in the same treatment group, and
(iii) tissue protein mass is the mass of protein in the individual dissected tissues from each animal when synthesis is measured.

Calculations of fractional degradation rate (K_deg_)

For each individual rat muscle, K_deg_ is obtained by subtracting K_growth_ from K_syn_;

$$\begin{array}{l} \text{i}.\text{e}.,\;{\text{K}}_{\text{deg}}\;\left(\text{\%}/\text{d}\right)\;={\text{K}}_{\text{syn}}\;\left(\text{\%}/\text{d}\right)—{\text{K}}_{\text{growth}}\;\left(\text{\%}/\text{d}\right). \end{array}$$

Inherent in this calculation that provides an estimate of protein degradation are the following assumptions: (i) over the days interval at which the growth of the rat was measured, the growth of the protein mass in the muscle was proportional to that of the whole body, and (ii) the rate of muscle protein synthesis measured over 50 min (incorporation time period between injection of L-[^13^C]-valine and animal sacrifice), is similar to the average rate for the days period over which time the growth rate is measured. These assumptions have been validated in actively growing rats (30–32), and this method of *in-vivo* determination of protein turnover in skeletal muscle has been utilized under a variety of nutritional and environmental conditions (29–36).

### Net T3 Neogenesis Assay

The kinetics of thyroid hormone metabolism in skeletal muscle were assessed *in vitro* as described previously (18), using the method of Kaplan and Utiger (37) by incubating muscle homogenates in Tris buffer at 37°C. The T3 neogenesis reaction was started by adding T4 (1.3 μM) dissolved in PBS containing 0.25% BSA. Aliquots of the homogenate were removed after 0, 5, 10, 15, and 30 min, the reaction was stopped by adding 95% ethanol, and the samples were stored at 4°C until assayed for thyroid hormone content using T3/T4 enzyme immunoassay kits (from Diagnostic System Laboratories, Webster, Texas, USA).

### Protein Extraction and Western Blotting

The expression levels of skeletal muscle DIO proteins were determined by Western blots according to standard procedures described in details elsewhere (38, 39). Hindlimb skeletal muscles (gastrocnemius, soleus, and tibialis anterior) were harvested and immediately put in liquid nitrogen. Frozen tissues (30 mg) were rapidly weighed and homogenized in liquid nitrogen. Muscle proteins were extracted in 9 volumes of Guba-Straub buffer (300 mM NaCl, 100 mM NaH_2_PO_4_, 10 mM Na_2_HPO_4_, 10 mM Na_4_P2O_7_, 1 mM MgCl_2_, 10 mM EDTA (pH 6.5) containing 0.1% 2-mercaptoethanol and 0.2% protease and phosphatase inhibitor cocktail. After incubation for 45 min at 4°C, samples were sonicated for 10 s, Triton X-100 (Applichem, Axon Lab AG, Le Mont-sur-Lausanne, Switzerland) was added to a final concentration of 1% and extracts were centrifuged at 12,000 g for 15 min at 4°C. The supernatants were collected, and protein content was determined using Bradford method (BioRad, California, USA). Extracts were first diluted in Guba-Straub buffer before the addition of Laemmli buffer at a final concentration of 3 mg/mL before being used for immunoblotting. Thirty micro gram of protein extract was separated by SDS-PAGE and blotted on PVDF membranes. Membranes were incubated first with primary antibodies (details in Table 1), and then secondary antibody LI-COR anti-rabbit (dilution 1/15,000) or anti-goat (dilution 1/15,000) were used to detect bands. The signals were visualized and quantified with the use of Odyssey Infrared Imaging System (Li-Cor Biosciences, Bad Homburg, Germany), and normalized with Ponceau Red. Validation of the antibodies used for detecting and quantifying DIO1, DIO2, and DIO3 has been detailed as supplementary material in a previous report (18). For each DIOs, between-group comparisons were performed separately at each of the following two time-points: (i) at the end of semistarvation (SS group) vs. the controls (Css group) and (ii) after 7 days of refeeding (RF group) vs. their controls (C_RF_ group). Each between-group comparison (SS vs. Css or RF vs. C_RF_) for a given muscle was thus made on the same gel and under the same conditions (sample preparation, exposure conditions toward antibodies, etc.).

**Table 1:** Primary antibodies and specific conditions used for analysis of DIO protein levels.

	**DIO1**	**DIO2**	**DIO3**
Supplier	Proteintech Europe	Santa-Cruz biotechnology	Novus biological
Catalog number	11790-1-AP	sc-98716	NBP1-05767
Dilution used	1/1,000	1/200	1/1,000
Blocking buffer	BSA	Milk	Milk
Gel	0.8 mm	0.8 mm	0.8 mm
Stacking	4%	4%	4 %
Resolving	12%	12%	12 %
Running	50 V for 30 min	50 V for 30 min	50 V for 30 min
conditions	150 V for 2h	150 V for 2h	150 V for 2h
Transfer conditions	400 mA for 1 h 30	400 mA for 1 h 30	400 mA for 1 h 30

### Data Analysis and Statistics

All data are presented as means ± SE. Unpaired *t*-test was used to assess the effects of semistarvation (semistarved vs. control rats) and refeeding (refed vs. control rats) on the various parameters; statistical significance of differences are indicated as follows: ^*^p < 0.05; ^**^p < 0.01; ^***^p < 0.001. The statistical treatment of data was performed using the computer software STATISTIX, version 8.0 (Analytical Software, St. Paul, MN).

## Results

### Body Weight and Body Composition

**Figure 1:**
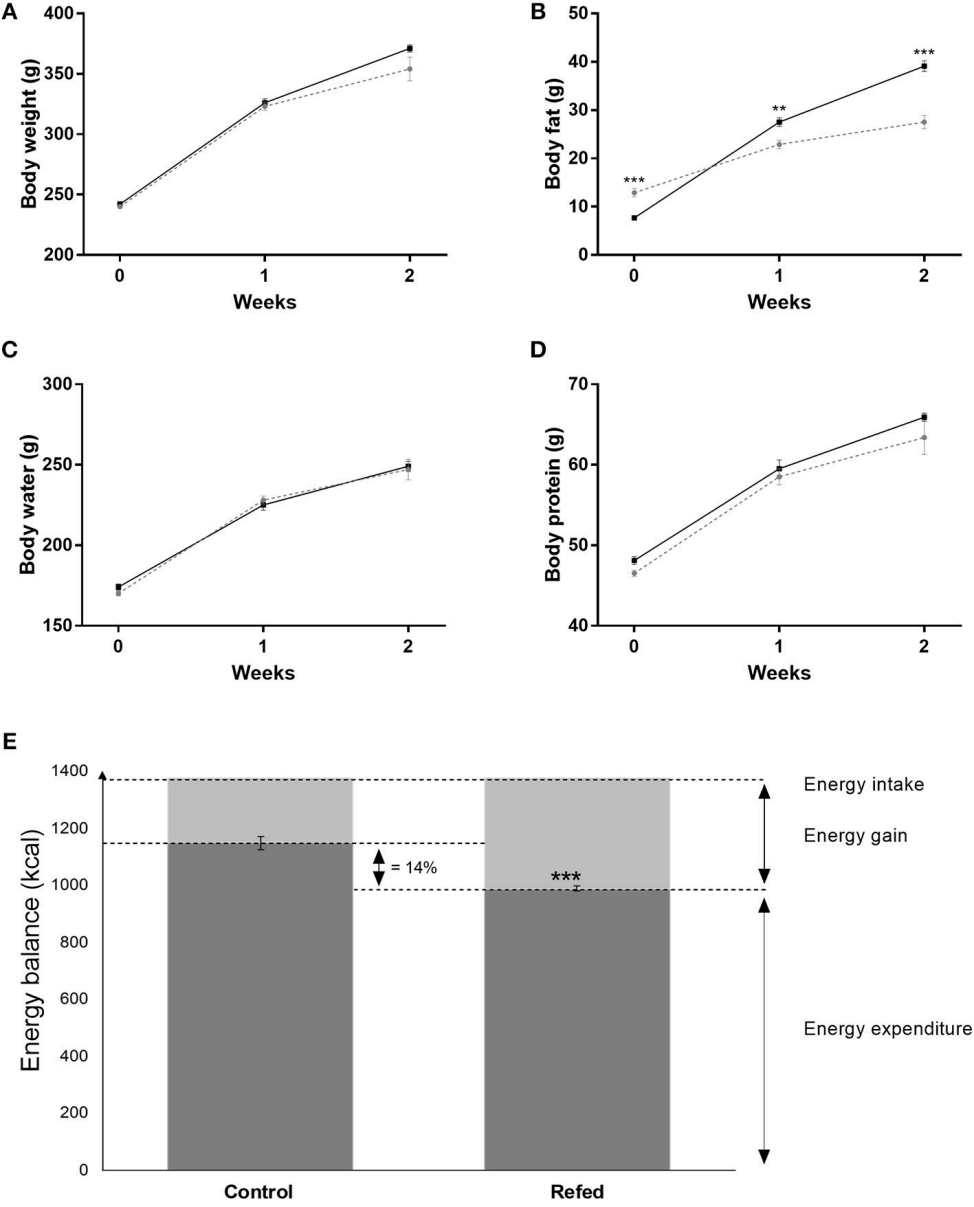
Body weight **(A)**, body water **(B)**, body fat **(C)**, and body protein **(D)** at the end of semistarvation (day 0 of refeeding) and at week 1 and week 2 of refeeding; the refed are shown in black lines and the control animals in gray (broken) lines. The bar charts **(E)** show the data on energy balance (energy intake, energy gain and energy expenditure) over the entire 2-week refeeding period for the refed and control groups. The values are mean ± SE (*n* = 6). Statistical significance of differences are indicated as follows: ^**^p < 0.01; ^***^p < 0.001.

The results on body weight and body composition are shown in Figures 1A–D. As previously reported (3, 4), the refed animals gained body fat faster than the controls during both week 1 and 2 of isocaloric refeeding (Figure 1B), whereas the gain in lean (protein) mass was not different (Figure 1D). The data on energy balance, body energy gain and total energy expenditure, shown in Figure 1E (as bar charts) indicate that over the 2-week period of refeeding, the total energy expenditure was lower in refed animals than in the controls (−14%, *p* < 0.001); the latter underlying the phenomenon of energy conservation directed at accelerating fat deposition during weight recovery.

### Skeletal Muscle Protein Synthesis and Protein Turnover

**Figure 2:**
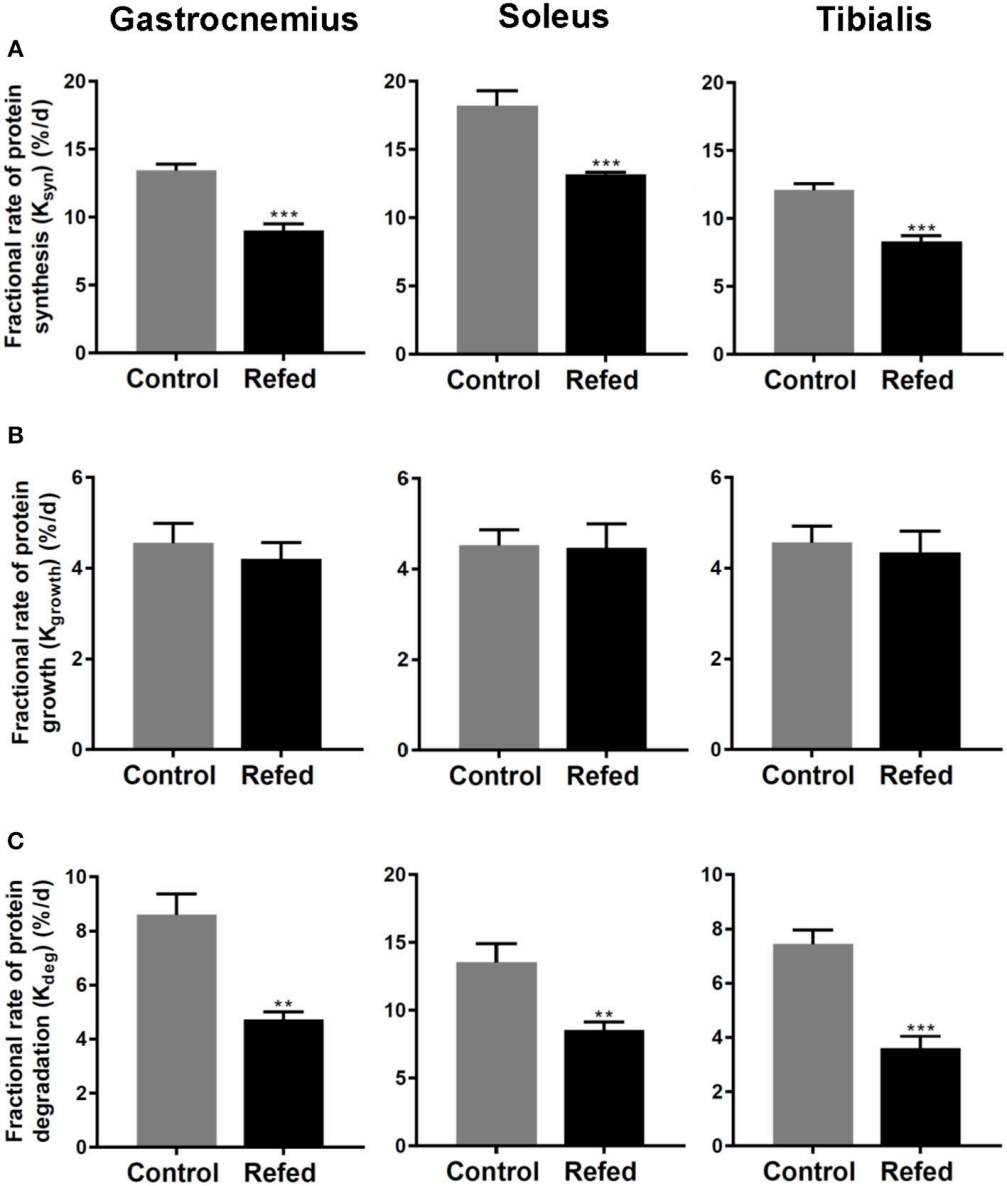
Fractional rates of **(A)** protein synthesis (K_syn_), **(B)** protein growth (K_growth_), and **(C)** protein degradation (K_deg_) in the Gastrocnemius, Soleus and Tibialis anterior muscles of rats after 7 days of refeeding in refed and control groups. The values are mean ± SE (*n* = 6–7). Statistical significance of differences are indicated as follows: ^**^p < 0.01; ^***^p < 0.001.

The results of fractional synthesis rate (K_syn_) of proteins assessed *in vivo* in the hindlimb skeletal muscles are shown in Figure 2A. K_syn_ was significantly lower in all three skeletal muscles from refed animals than from controls, namely by 33% (*p* < 0.001) in the gastrocnemius, by 28% (*p* < 0.001) in the soleus, and by 31% (*p* < 0.001) in the tibialis anterior. By contrast, in all 3 skeletal muscles studied, there was no difference in the fractional growth rate (K_growth_) between the refed and control groups (Figure 2B). From the data on K_syn_ and K_growth_, the calculated fractional protein degradation rate (K_deg_) was found to be significantly lower in the refed than the control animals in all three muscles (Figure 2C). Thus, in the absence of between-group differences in fractional growth rate, the lower fractional protein synthesis as well as degradation rates in the refed animals than in the controls suggest that the refed animals show diminished rate of protein turnover in all the three muscles studied during the phase of catch-up fat.

### Net T3 Neogenesis

**Figure 3:**
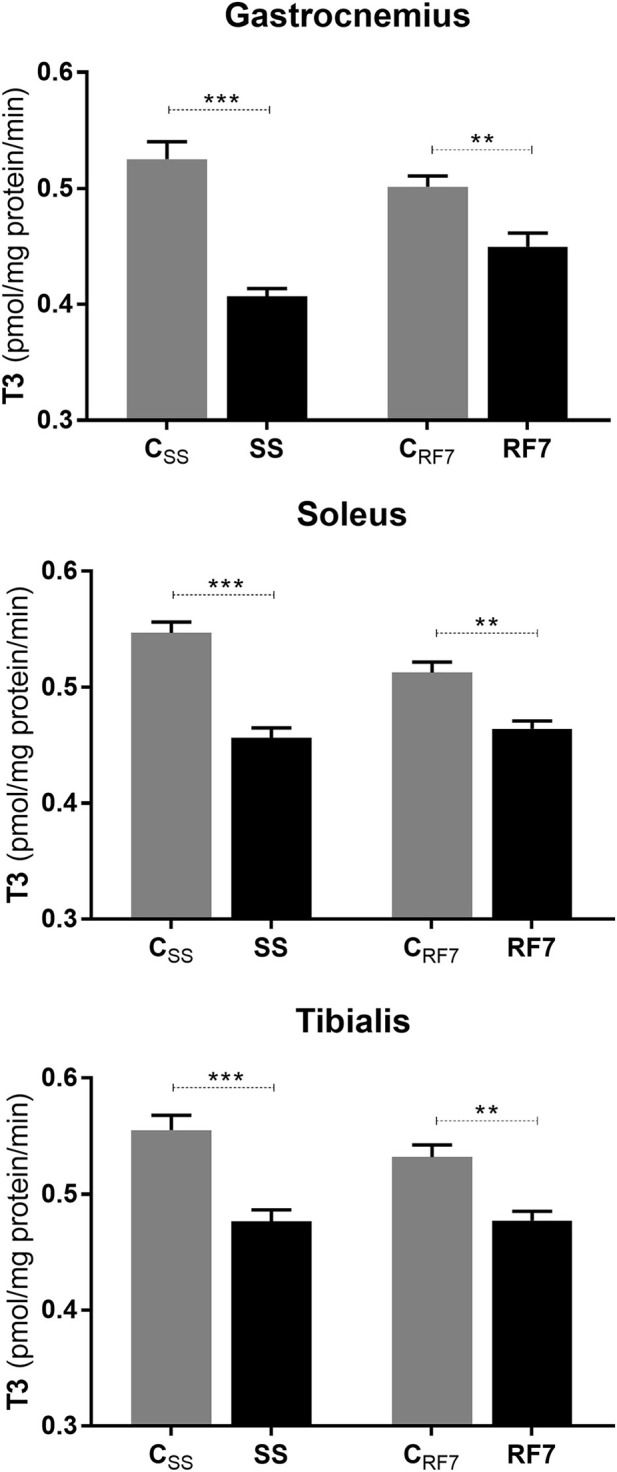
Net T3 formation from its T4 precursor in Gastrocnemius, Soleus and Tibialis anterior muscles from (i) rats semistarved (SS) for 14 days and their controls (C_SS_), and (ii) rats refed for 7 days (RF7) and their controls (C_RF7_). The values are mean ±SE (*n* = 6). Statistical significance of differences are indicated as follows: ^**^p < 0.01; ^***^p < 0.001.

The data on the rate of net T3 neogenesis, assessed *ex vivo* in extracts of the gastrocnemius, soleus and tibialis anterior muscles from rats at the end of semistarvation (SS group) and their controls (C_SS_), as well as and after 7 days of refeeding (RF7 group) and their controls (C_RF7_), are presented in Figure 3. The *de novo* net T3 synthesis was found to be significantly lower in muscles of semistarved rats than in controls, namely by −22, −17, and −14% in gastrocnemius, soleus and tibialis anterior, respectively (*p* < 0.001). After 7 days of refeeding, it was still significantly lower in all three muscles from refed rats than in the controls (by about −10%, *p* < 0.01) in all the muscle types.

### Deiodinases Protein Expression

**Figure 4:**
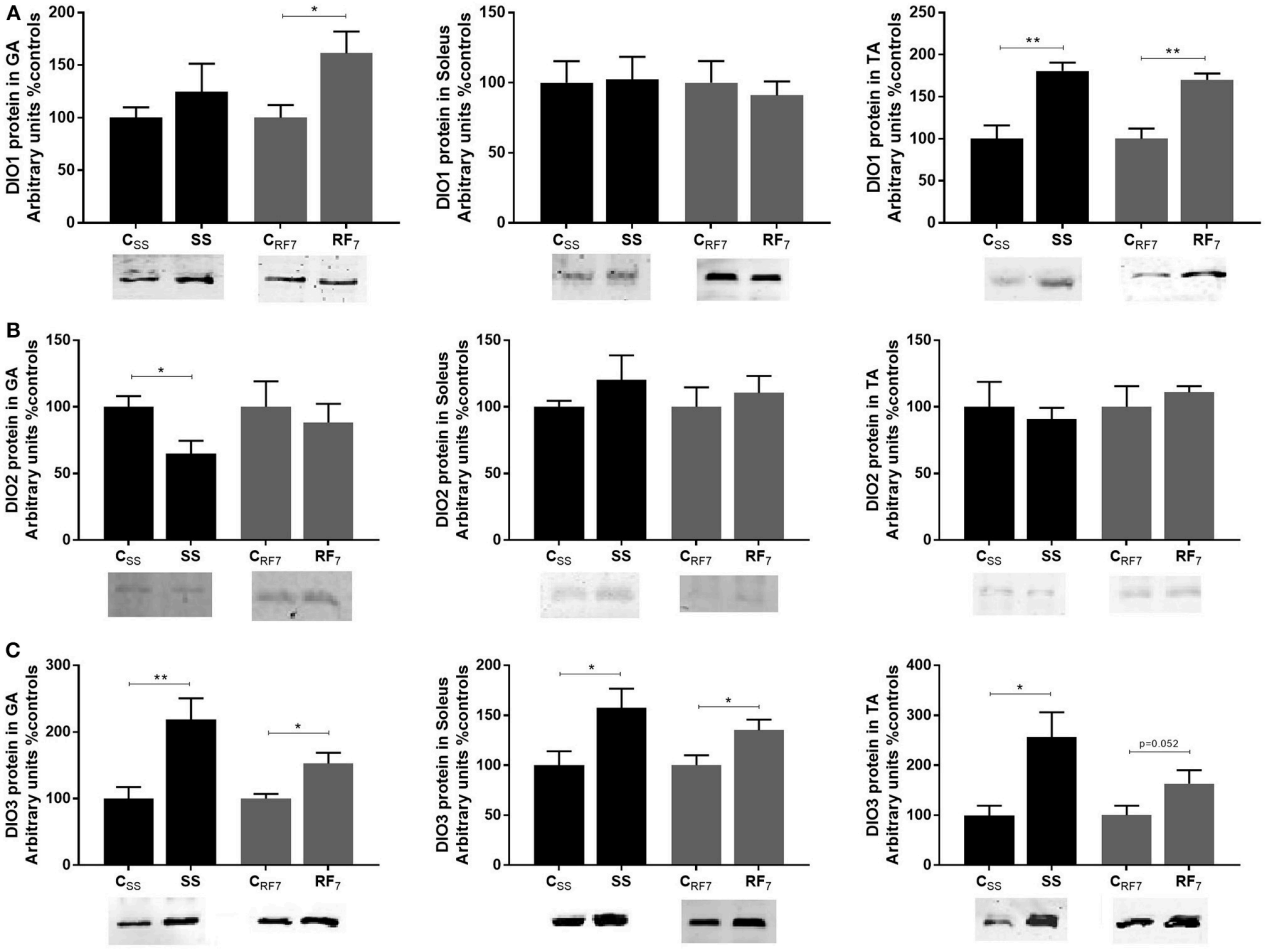
Protein expression of the deiodinases: **(A)** DIO1, **(B)** DIO2, and **(C)** DIO3 in Gastrocnemius (GA), Soleus and Tibialis anterior (TA) muscles from (i) rats semistarved (SS) for 14 days and their controls (C_SS_), and (ii) rats refed for 7 days (RF7) and their controls (C_RF7_). The values are mean ±SE (*n* = 4). Statistical significance of differences are indicated as follows: ^*^p < 0.05; ^**^p < 0.01.

As net T3 neogenesis is the outcome of T3 synthesis from T4 (through deiodinization catalyzed primarily by DIO2) and T3 degradation (by DIO3 and to a lesser extent by DIO1) (40), we investigated the expression of these deiodinases in all three muscles. The results showing the abundance of the deiodinase proteins (DIO1, DIO2, DIO3) at the end of semistarvation and after 1 week of refeeding are shown in Figure 4. For DIO1 (panel A), it was more abundant in the tibialis anterior of both semistarved (SS) and refed (RF) rats relative to their respective controls (by about 75%, *p* < 0.01), and to a lesser extent in the gastrocnemius muscle, namely about +25% in SS rats (non-significant) and about +60% in RF rats (*p* < 0.05). DIO1 abundance in the soleus muscle was not different in SS or RF rats relative to their respective controls. For DIO2 (Figure 4B), there was a significant difference in its abundance only in the gastrocnemius muscle of SS rats than in controls (lower by 35%, *p* < 0.05), but not in the RF rats relative to controls. Furthermore, the abundance of DIO2 in the two other muscles (soleus and tibialis anterior) was not different in SS and RF rats relative to their respective controls. By contrast, DIO3 (panel C) was more abundant in all three muscles from the SS than in the controls, namely by 2.2- and 2.5-fold higher in gastrocnemius and tibialis anterior, respectively, and by about 60% higher in soleus. Although less pronounced than during semistarvation, the abundance of DIO3 in all three skeletal muscles after 1 week of refeeding was also higher in refed animals than in controls: namely +53, +35, and +63% in gastrocnemius, soleus, and tibialis anterior, respectively, with the difference being statistically significant in the gastrocnemius and soleus muscles (*p* < 0.05).

## Discussion

The results presented here suggest a role for diminished protein turnover in skeletal muscle, associated with altered muscle thyroid hormone metabolism resulting in diminished T3 availability, in the high efficiency with which body fat is recovered after substantial body fat depletion.

### Diminished Rate of Protein Synthesis and Turnover

Using the stable isotope flooding dose technique of incorporation of ^13^C-labeled valine in protein for *in-vivo* measurements of protein synthesis in skeletal muscle, the fractional protein synthesis rate (K_syn_) is shown to be lower in skeletal muscle of refed animals than in controls assessed on day 7 of refeeding, i.e., at about mid-point in the dynamic phase of catch-up fat. The lower muscle K_syn_ in refed animals was observed in all three hindlimb skeletal muscles studied, namely the gastrocnemius which is predominantly fast-oxidative glycolytic, soleus which is predominantly slow-oxidative and the tibialis anterior which is predominantly glycolytic, thereby suggesting that the lower K_syn_ during catch-up fat occurs independently of skeletal muscle fiber composition. As the rate of skeletal muscle growth (K_growth_) was similar in refed and control animals (in line with similar rates in total body protein deposition), it follows that the reduced rate of muscle protein synthesis is accompanied by reduced rate of muscle protein degradation (K_deg_), thereby underscoring a role for diminished protein turnover in skeletal muscle in the suppressed thermogenesis that drives catch-up fat.

Diminished skeletal muscle K_syn_ in the postabsorptive state has previously been shown in obese humans (41) and in rats made obese on a high-fat high-sucrose diet (26), and is considered to reside in impaired amino acid incorporation into proteins attributed to lipid infiltration and insulin resistance in the skeletal muscle (42). These authors have proposed that the mechanisms underlying such reductions in muscle protein synthesis may reside in an inhibitory effect of lipid derivative species on insulin signaling which would result in diminished protein translation, and that insulin resistance in skeletal muscle not only impairs glucose metabolism but also protein metabolism (42). Although skeletal muscle insulin resistance has been shown to be an early event that is sustained throughout the dynamic phase of catch-up fat in our rat model (43, 44), the lower K_syn_ in skeletal muscle is reported here at a time-point of refeeding (day 7) when (i) skeletal muscle lipid content was not found to be higher in muscles from refed animals than in controls (17, 43), and also (ii) when total and regional body fat content of the refed animals had not yet exceeded those of controls (17, 43, 44). Furthermore, the findings here of diminished K_syn_ in all muscle types varying widely in fiber composition during catch-up fat contrast with past reports of muscle fiber-type dependency of the lower K_syn_ observed in obese rats, namely only in glycolytic muscles but not in the soleus (slow-oxidative) muscle of diet-induced obese rats (26), or only in red oxidative fibers and not in white glycolytic fibers of the gastrocnemius of the genetic (leptin receptor deficient) obese Zucker rats (45). Taken together, therefore, the lower K_syn_ observed here in skeletal muscle during catch-up fat is unlikely to be a consequence of excess whole body, regional or lipid infiltration in skeletal muscle, but rather reflects a diminished rate of substrate cycling between protein synthesis and degradation (i.e., reduced protein turnover) for the purpose of sparing energy for catch-up fat.

### Altered Skeletal Muscle Thyroid Hormone Metabolism

As the thyroid hormone T3 is known to play an important role in the control of basal metabolism and thermogenesis (13–15), we have in past studies examined the extent to which the well-known reduction in circulating levels of T3 during caloric restriction is restored during refeeding in the rat model of catch-up fat (16, 17). In particular, we found that while the blood concentrations of TSH, T4 and T3 are all markedly lower at the end of caloric restriction, refeeding resulted in differences in their restoration kinetics. Indeed, whereas plasma TSH and T4 were completely restored to control levels by day 5 of refeeding, plasma T3 remained lower, albeit marginally, in the refed animals than in controls up to day 10 day of refeeding (16), and could hence contribute to the diminished thermogenesis driving catch-up fat.

However, circulating levels of thyroid hormones may not necessarily reflect tissue thyroid hormone levels as the bioavailability at the tissue and cellular level is dependent upon local thyroid hormone metabolism (14, 15, 40). The net formation of T3 from T4 (i.e., net T3 neogenesis) is to a large extent controlled by an interplay of deiodinase enzymes that catalyze activation or inactivation of T4 and T3. In the skeletal muscle, the conversion of T4 into the active hormone T3 is believed to be primarily catalyzed by DIO2 and the inactivation of T4 and T3 to be catalyzed by DIO3 (and possibly also by DIO1) to rT3 and T2 (3,3′diiodothyronine) (40). In a recent study from our laboratory (18) investigating possible alterations in thyroid hormone metabolism in skeletal muscle during catch-up fat, it was reported that the *in-vitro* kinetics of T3 generation in the T4-incubated gastrocnemius muscle of semistarved and refed rats are significantly lower than in their respective controls. Explanations based upon altered deiodinase activities were reinforced by the findings that the protein expression DIO2 was reduced while that of DIO3 was increased in this hindlimb muscle both during caloric restriction and refeeding (18).

In the present study, we have extended these investigations to other muscle types varying widely in fiber composition. Using the same *in-vitro* kinetic assay of T3 generated by T4 in incubated skeletal muscle, we show that the reduction in net T3 neogenesis in muscle during semistarvation and refeeding is observed not only in the gastrocnemius muscle, but also in the soleus and tibialis anterior, thereby suggesting that the reduction in muscle T3 availability during caloric restriction and persisting during the catch-up fat phase occurs independently of skeletal muscle fiber composition, and may involve the whole skeletal muscle mass. By contrast, alterations in the abundance of the three deiodinases are found to vary according to muscle type. In response to semistarvation and after 1 week of refeeding, DIO2 which is considered to be primarily responsible for T3 production from T4 in skeletal muscle was less abundant in the gastrocnemius muscle, but not in the soleus or tibialis anterior. The abundance of DIO1 (which may limit T3 availability by diverting T4 and T3 to inactive rT3 and T2) was higher in the gastrocnemius and tibialis anterior but not in the soleus. The most striking feature in the analysis of these data on deiodinases is the robust upregulation of DIO3 observed in all 3 muscle types during semistarvation, which persisted after 1 week of refeeding in two of these 3 muscles, namely in the gastrocnemius and soleus. Interestingly, the abundance of DIO1 in the tibialis anterior, which was increased during semistarvation, also persisted during refeeding (+70% relative to controls, *p* < 001). Thus, during refeeding, in the absence of a robust increase in the abundance of the T3 inactivator DIO3 in the tibialis anterior, the increased DIO1 in this muscle may assume a greater importance than DIO3 upregulation in reducing T3 availability. Taken together, our results suggest that the lower net T3 neogenesis in all three muscles of varied fiber composition studied during semistarvation and catch-up fat seems to reside primarily in the upregulation of the thyroid hormone inactivating enzymes DIO1 and/or DIO3 (i.e., deiodinases that catalyze the conversion of T4 and T3 to biologically inactive rT3 and T2) rather than in the downregulation of the thyroid hormone activating enzyme DIO2 which catalyzes the conversion of T4 to T3. A better understanding of how the muscle-type dependent changes in these DIOs are co-ordinated to result in diminished T3 availability during semistarvation and refeeding will need to be addressed in future studies involving the use of sensitive assays to detect the changes in the activity of these three deiodinases in rat skeletal muscle.

Whatever, the mechanisms controlling the upregulation of the T3 inactivating deiodinase enzymes in the various skeletal muscle types, our findings here indicate that, the kinetics of T3 generation in skeletal muscle homogenates incubated with T4 were lower in semistarved and refed rats. This underscores the possibility that a lower T_3_ availability in skeletal muscle during semistarvation and refeeding could be contributed not only from a lower plasma T_3_ level (16, 17), but also from altered muscle deiodinase activities. Given the role of T3 in controlling many inter-related aspects of skeletal muscle energetics that include the maintenance of ionic equilibrium through Na/K ATPase, calcium cycling, fiber composition, contraction-relaxation kinetics, protein synthesis, and protein turnover, the relative hypothyroidism in skeletal muscle during semistarvation and persisting during refeeding may thus contribute to the suppression of thermogenesis during caloric restriction and subsequent high efficiency for catch-up fat.

## Conclusion

The results presented here suggest that diminished skeletal muscle protein turnover, together with altered local muscle metabolism of thyroid hormones leading to diminished intracellular T3 availability, are features of the thrifty metabolism that drive the rapid restoration of the fat reserves during weight regain after caloric restriction.

## Author Contributions

AD, JC, YB, and CG conceived and designed the experiments. JC, LI, JM-C, DA, and CG performed the experiments. JC, AD, DA, CG, and YB analyzed the data. AD, J-PM, CG, and YB contributed reagents, materials, and analysis tools. JC and AD wrote the paper. LI, JM-C, DA, J-PM, CG, and YB edited the manuscript.

### Conflict of Interest Statement

The authors declare that the research was conducted in the absence of any commercial or financial relationships that could be construed as a potential conflict of interest.
